# Association of Total Flavonoid Intake with Hypo-HDL-Cholesterolemia among Korean Adults: Effect Modification by Polyunsaturated Fatty Acid Intake

**DOI:** 10.3390/nu12010195

**Published:** 2020-01-10

**Authors:** Seoeun Ahn, Shinyoung Jun, Hyojee Joung

**Affiliations:** 1 Department of Public Health Science, Graduate School of Public Health, Seoul National University, Seoul 08826, Korea; ase0821@snu.ac.kr; 2 Department of Nutrition Science, Purdue University, West Lafayette, IN 47907, USA; jun24@purdue.edu; 3 Institute of Health and Environment, Seoul National University, 08826 Seoul, Korea

**Keywords:** flavonoids, polyunsaturated fatty acids, HDL-cholesterol, Korea National Health and Nutrition Examination Survey

## Abstract

The aim of this study was to examine the independent association between flavonoid intake and hypo-high-density lipoprotein (HDL)-cholesterolemia and the potential modifying effect of polyunsaturated fatty acid (PUFA) intake on this association among Korean adults. This cross-sectional analysis used data from 10,326 subjects who participated in the 2013–2016 Korean National Health and Nutrition Examination Survey. Multiple logistic regression analyses were used to assess the associations of flavonoid and PUFA intakes with hypo-HDL-cholesterolemia prevalence. Proanthocyanidins intake showed an inverse relationship with hypo-HDL-cholesterolemia only in men (Tertile (T) 3 vs. T1: odds ratio (OR) = 0.74, 95% confidence interval (CI) = 0.59–0.92, *p*-trend = 0.0330). Total flavonoid and PUFA intakes were not associated with hypo-HDL-cholesterolemia in both men and women. However, when stratified by PUFA intake, there was an inverse relationship between total flavonoid intake and hypo-HDL-cholesterolemia prevalence in men with a high *n*-3 PUFA intake (total flavonoid intakes T3 vs. T1: OR = 0.59, 95% CI = 0.42–0.82, *p*-trend = 0.0004) or a low *n*-6/*n*-3 PUFA intake ratio (T3 vs. T1: OR = 0.67, 95% CI = 0.48–0.93, *p*-trend = 0.0053), but not in those with a low *n*-3 PUFA intake (*p*-interaction = 0.0038) or a high *n*-6/*n*-3 PUFA intake ratio (*p*-interaction = 0.1772). In women, no association was found between total flavonoid intake and hypo-HDL-cholesterolemia, regardless of PUFA intake. These results imply that the intake of proanthocyanidins might have beneficial effects on the HDL-cholesterol level in Korean men. In addition, *n*-3 PUFA intake might modify the association of total flavonoid intake with the hypo-HDL-cholesterolemia among Korean men.

## 1. Introduction

The World Health Organization Global Burden of Diseases report estimated that 17,858,000 people died of cardiovascular diseases (CVD) in 2016, accounting for 31.4% of all deaths worldwide [1]. In South Korea, 119.6 deaths out of every 100,000 people in 2017 were attributed to CVD, including heart disease, cerebrovascular disease, and hypertensive disease [2]. High-density lipoprotein (HDL)-cholesterol is a well-known protective factor against CVD as HDL-cholesterol can lower the risk of atherosclerosis through multiple pathophysiologic mechanisms, such as reverse cholesterol transport and antioxidant and anti-inflammatory activities [3,4,5].

Flavonoids, phytochemicals rich in fruits and vegetables, are known to have antioxidant and anti-inflammatory effects that have the potential to increase HDL-cholesterol levels, thus, reducing the risk of CVD [6,7]. A recent meta-analysis of 15 cohort studies concluded that there is an inverse association between flavonoid intake and CVD-associated mortality [8]. A recent systematic review of randomized controlled trials (RCT) also demonstrated the positive effects of the intakes of flavonoid supplements, extracts, flavonoid-rich foods, and flavonoid-fortified foods on both the levels and functions of HDL-cholesterol [9].

Dietary polyunsaturated fatty acids (PUFA), particularly omega-3 (*n*-3) PUFA, have also been extensively studied for their antiatherogenic effects [10,11], which seem to be mediated by their anti-inflammatory properties [12]. Accordingly, *n*-3 PUFA may interact with the antioxidant or anti-inflammatory properties of flavonoids, which may have an impact on the association between flavonoids and HDL-cholesterol. In fact, in animal studies, the intake of total flavonoids or PUFAs improved each other’s bioavailability [13,14,15]. However, few human studies have investigated the effect of PUFA intake on the association between flavonoid intake and HDL-cholesterol levels.

Many studies have consistently observed a higher incidence of CVD in men compared to women [16,17,18]. In general, CVD develops about 10 years later in women than in men [19]. In addition, women have higher HDL-cholesterol levels than men [20,21]. According to the Korean Society of Lipid and Atherosclerosis, the prevalence of dyslipidemia among Korean adults aged 30 years and older was 47.9% for men and 34.4% for women. Especially for hypo-HDL-cholesterolemia, defined as serum HDL-cholesterol <40 mg/dL, the prevalence in men (≥30 years) was 27.2%, more than twice that in women (11.8%) [22]. Thus, CVD and its risk factors need to be investigated in a sex-specific manner.

The primary objective of our study was to investigate whether the dietary intakes of flavonoids (total flavonoid, flavonols, flavones, flavanones, flavan-3-ols, isoflavones, anthocyanidins, and proanthocyanidins) or PUFAs (total PUFA, *n*-6 PUFA, *n*-3 PUFA, and *n*-6/*n*-3 PUFA ratio) were associated with hypo-HDL-cholesterolemia among Korean men and women. Our secondary aim was to assess whether the dietary intake of PUFAs from foods modify the association of total flavonoid intake with hypo-HDL-cholesterolemia among Korean men and women.

## 2. Materials and Methods

### 2.1. Study Design and Subjects

The Korean National Health and Nutrition Examination Survey (KNHANES) is a nationally representative cross-sectional survey that has been used to assess health status, health behaviors, and food and nutrient intakes of the Korean population since 1998. A detailed description of the KNHANES can be found elsewhere [23]. This study used data obtained from the 2013–2016 KNHANES, as 2013 was the first year to release individual PUFA intake information. The data collection process was approved by the Institutional Review Board of the Korea Centers for Disease Control and Prevention, and informed consent was obtained from every participant. Secondary analyses of data for this study were approved by the Institutional Review Board of the Seoul National University (IRB No. E1905/003-008).

Of the 15,797 participants aged 19–64 years who completed a 24-h recall survey, we excluded participants according to the following criteria: missing data on height, weight, or fasting HDL-cholesterol (*n* = 1690); fasting < 8 h prior to blood collection (*n* = 305); pregnant or lactating women (*n* = 187); self-reporting diagnosis of hypertension, dyslipidemia, diabetes, or CVD (stroke, myocardial infarction, or angina pectoris) or current use of medications for hypertension, dyslipidemia, or diabetes (*n* = 3078); or having an extremely high (≥5000 kcal/d) or low (<500 kcal) daily energy intake (*n* = 211). Finally, 10,326 participants (4061 men and 6265 women) were included in the final analyses.

### 2.2. Data Collection

In KNHANES, trained interviewers conducted a 24-h recall interview to determine the types and amount of all foods consumed by the subjects during the past 24 h. Individual daily intakes of energy, carbohydrate, protein, fat, total PUFA, *n*-6 PUFA, and *n*-3 PUFA were calculated in the KNHANES using the Korean foods composition table. Individual flavonoid intakes were estimated by combining food consumption data with a flavonoid database of common Korean foods, which is based on the Korea Functional Food Composition Table, US Department of Agriculture flavonoid database for selected foods, and the Phenol-Explorer database [24]. The daily total flavonoid intake was determined by the summation of the seven major flavonoid subclasses (flavonols, flavones, flavanones, flavan-3-ols, isoflavones, anthocyanidins, and proanthocyanidins) intakes.

Information regarding the participants’ sociodemographic (household income and education level) and lifestyle factors (alcohol consumption, smoking status, and physical activity) were obtained from the KNHANES health interviews. The household income levels were divided into quartiles according to the monthly average household income [monthly household income/√ (number of family members)], and the first, second, third, and fourth quartiles corresponded to low, middle-low, middle-high, and high incomes, respectively. The education level was classified into three groups (middle school or less, high school, or college or higher). Alcohol consumption level of subjects was classified according to the frequency and amount, such as none or low (less than once per month during the last year), moderate (less than twice per week and an average consumption per time of <7 cups for men and <5 cups for women), high (more than once per month but not moderate nor very high drinker), or very high (more than twice per week and an average consumption per time of ≥7 cups for men and ≥5 cups for women). Smoking status was classified into three groups: never (never smoked cigarettes or <100 cigarettes during lifetime), former (smoked ≥100 cigarettes during lifetime but currently non-smoking), or current (smoked ≥100 cigarettes during lifetime and currently smoking). Physical activity was recorded as “yes” for individuals performing activities per week: a vigorous intensity activity for >75 min, a moderate intensity activity for >150 min, or an equivalent activity combining both types.

Anthropometric measurements and laboratory tests were performed during the health examination section of the KNHANES. Detailed descriptions of investigation methods are presented on the official KNHANES website [25]. Anthropometric data, including height and weight, were measured by trained health technicians using calibrated equipment and standardized protocols. Body mass index (BMI) was calculated by dividing body weight (kg) with the square of height (m^2^), and obesity status was classified as underweight (<18.5 kg/m^2^), normal weight (18.5 to <25 kg/m^2^), or obese (≥25 kg/m^2^). Blood samples were collected from the participants after a fasting period of ≥8 h. Fasting serum total cholesterol, HDL-cholesterol, and triglyceride levels were measured using a Hitachi Automatic Analyzer 7600 (Hitachi Co., Tokyo, Japan). For participants with triglyceride levels of <400 mg/dL, the low-density lipoprotein (LDL)-cholesterol were calculated using Friedewald’s formula: (LDL-cholesterol) = (total cholesterol) − (HDL-cholesterol) − (triglyceride/5) [26]. Men with an HDL-cholesterol level <40 mg/dL and women with an HDL-cholesterol level <50 mg/dL were classified to have hypo-HDL-cholesterolemia according to the National Cholesterol Education Program Adult Treatment Panel III criteria [27].

### 2.3. Statistical Analysis

The dietary intakes of flavonoids (total flavonoid, flavonols, flavones, flavanones, flavan-3-ols, isoflavones, anthocyanidins, and proanthocyanidins), PUFAs (total PUFA, *n*-6 PUFA, and *n*-3 PUFA), and macronutrients (carbohydrate, protein, and fat) were adjusted for the total energy intake using the residual method for men and women [28]. The participants were divided into tertiles according to the flavonoid or PUFA intakes by sex. Differences in general characteristics across the total flavonoid intake tertiles were assessed using the analysis of variance (ANOVA) for continuous variables and the chi-square test for categorical variables.

Multiple logistic regression analyses were used to estimate the odds ratios (OR) and 95% confidence intervals (95% CI) for hypo-HDL-cholesterolemia according to the flavonoid and PUFA intake tertiles. The lowest intake categories were used as the reference. ORs and 95% CIs were adjusted for age (continuous), BMI (<18.5, 18.5–24.9, or ≥25 kg/m^2^), serum triglyceride levels (continuous), alcohol consumption (none or low, moderate, high, very high, or missing), smoking status (never, former, current, or missing), household income (low, middle-low, middle-high or missing, or high), education level (≤middle school, high school, or ≥college), and energy intake (continuous). To determine whether the PUFA intake modified the relationship between the total flavonoid intake and the prevalence of hypo-HDL-cholesterolemia, we also conducted analyses stratified by PUFA intakes; here, the PUFA intake was dichotomized (less than/greater than or equal to the median intake) to ensure the inclusion of sufficient numbers of hypo-HDL-cholesterolemia cases in each stratum. To calculate the *p* for interaction values, we conducted further multiple logistic regression analyses that included all the above-listed variables and additional cross-product terms between the total flavonoid and PUFA intakes.

All statistical analyses were performed using SAS 9.4 (SAS Institute, Cary, NC, USA). The analyses were reflective of the complex sampling design of KNHANES. All tests were two-sided, and a *p*-value < 0.05 was considered statistically significant.

## 3. Results

### 3.1. General Characteristics of the Study Subjects

Table 1 presents the general characteristics of the study subjects according to the total flavonoid intake tertiles. Both men and women who consumed more total flavonoids were significantly more likely to be older, have higher household income level, and were less likely to be high alcohol drinkers and current smokers (all *p* < 0.0001). Men who consumed more total flavonoid had a higher education level (*p* < 0.0001), but the education level of female subjects was not different according to the total flavonoid intake. Women who consumed more total flavonoid had a lower percentage of obesity than women who consumed less total flavonoid (*p* = 0.0084). With regard to nutrient intake, the higher the intake of total flavonoids, the lower were the intakes of energy and fat and higher were the intakes of carbohydrate and *n*-3 PUFA in both sexes (all *p* < 0.01). The total cholesterol and LDL-cholesterol levels of the male and female participants who consumed more total flavonoids were higher than those who consumed less total flavonoids (all *p* < 0.05). There were no significant differences in triglycerides and HDL-cholesterol levels across total flavonoid intake tertile in both men and women.

### 3.2. Association between Flavonoid or PUFA Intakes with Hypo-HDL-Cholesterolemia

Association between the intakes of flavonoids and PUFAs with hypo-HDL-cholesterolemia is shown in Table 2; Table 3. Table 2 presents the ORs and 95% CIs for hypo-HDL-cholesterolemia according to the flavonoid and PUFA intake tertiles. The dietary intakes of total flavonoid, total PUFA, *n*-6 PUFA, and *n*-3 PUFA, and the intake ratio of *n*-6/*n*-3 PUFA were not found to be associated with hypo-HDL-cholesterolemia in both men and women. Analyses of the association between intakes of flavonoid subclasses and hypo-HDL-cholesterolemia showed that dietary proanthocyanidins inversely associated with hypo-HDL-cholesterolemia in male subjects (T3 vs. T1: OR = 0.74, 95% CI = 0.59–0.92, *p*-trend = 0.0330).

Table 3 presents data regarding the effect modification of PUFA intake (total PUFA, *n*-6 PUFA, *n*-3 PUFA, and *n*-6/*n*-3 PUFA intake ratio) on the association between the dietary total flavonoid intake and hypo-HDL-cholesterolemia. An inverse relationship between total flavonoid intake and hypo-HDL-cholesterolemia was significant in men with a high *n*-3 PUFA intake (T3 vs. T1: OR = 0.59, 95% CI = 0.42–0.82, *p* for trend = 0.0004), but not in men with a low *n*-3 PUFA intake (*p* for interaction = 0.0038). In analyses stratified by the dietary *n*-6/*n*-3 PUFA ratio, a significant inverse association between total flavonoid intake and hypo-HDL-cholesterolemia was observed only in men with a low *n*-6/*n*-3 PUFA intake ratio (T3 vs. T1: OR = 0.67, 95% CI = 0.48–0.93, *p* for trend = 0.0053), but not in men with a high *n*-6/*n*-3 PUFA intake ratio. However, this interaction of total flavonoid with *n*-6/*n*-3 PUFA intake ratio was not statistically significant (*p* for interaction = 0.1772). The dietary total PUFA (*p* for interaction = 0.5494) and *n*-6 PUFA (*p* for interaction = 0.8782) intakes did not seem to modify the association between the total flavonoid intake and the prevalence of hypo-HDL-cholesterolemia in men. Women did not show any significant relation of total flavonoid intake and hypo-HDL-cholesterolemia according to PUFA intakes.

Since the inverse association of proanthocyanidins with hypo-HDL-cholesterolemia was identified, we further analyzed the association between dietary proanthocyanidins and hypo-HDL-cholesterolemia according to PUFA intakes (Supplementary Table S1). Significant inverse association between proanthocyanidins intake and hypo-HDL-cholesterolemia was shown in men with a low *n*-6 PUFA intake (T3 vs. T1: OR = 0.72, 95% CI = 0.53–0.97, *p*-trend = 0.0935), or in men with a high *n*-3 PUFA intake (T3 vs. T1: OR = 0.66, 95% CI = 0.49–0.89, *p*-trend = 0.0120), or in men with a low *n*-6/*n*-3 PUFA intake ratio (T3 vs. T1: OR = 0.68, 95% CI = 0.50−0.93, *p*-trend = 0.0451) respectively, but not in men with a high *n*-6 PUFA intake, or in men with a low *n*-3 PUFA intake, or in men with a high *n*-6/*n*-3 PUFA intake ratio. However, all *p* for interaction values were not significant (all *p* for interactions >0.2), indicating that PUFA intakes did not significantly affect the association between dietary proanthocyanidins and hypo-HDL-cholesterolemia.

## 4. Discussion

In this study, we examined the effects of the flavonoid on hypo-HDL-cholesterolemia as well as the effect modification of PUFA intakes on this association in Korean adults. In men, a higher intake of proanthocyanidins was associated with a decreased prevalence of hypo-HDL-cholesterolemia. An inverse relationship between the total flavonoid intake and hypo-HDL-cholesterolemia was not observed, but the possibility of effect modification of *n*-3 PUFA intake on this association was observed in men. However, a significant association was not found in women.

Flavonoids show considerable potential for improving blood HDL-cholesterol levels due to their antioxidant and anti-inflammatory properties. In the previous RCT studies, dietary anthocyanidins intake was associated with increased HDL-cholesterol levels [29,30,31,32,33]. However, the doses or concentrations of anthocyanidins used in RCT studies were too high to be taken from foods. The results of the previous observational studies about the association between total flavonoid intake from foods and HDL-cholesterol were inconsistent. In the follow-up study with German, total flavonoid intake from fruit, vegetables, and juices during adolescence was associated with higher HDL-cholesterol levels only in men, not in women [34]. Cross-sectional studies of Iranians, Italians and Americans showed positive relationships between total flavonoid intakes and HDL-cholesterol levels [35,36,37], but no correlation was observed in studies with Chinese and Polish [38,39]. In the present study, we found no significant relationship between total flavonoid intake and hypo-HDL-cholesterolemia among Korean adults. Therefore, further studies are needed to determine how much flavonoids intake increases HDL-cholesterol level and which subclasses of flavonoids play a major role in increasing HDL-cholesterol.

Thus, we conducted further analyses on the association between intakes of flavonoid subclasses and hypo-HDL-cholesterolemia. Among the seven subclasses of flavonoids, dietary proanthocyanidins, which accounted for the largest proportion (26.9%) of total flavonoid intakes of the participants in this study, inversely associated with hypo-HDL-cholesterolemia only in male subjects. Previous animal studies have shown that proanthocyanidins increase HDL-cholesterol levels [40,41], but human studies using foods or extracts rich in proanthocyanidins have not yet demonstrated the positive effects on HDL-cholesterol levels [42,43,44,45]. Further studies are needed to understand better the role of proanthocyanidins on the lipid profiles and the prevention of CVD.

In this study, the intakes of total PUFA, *n*-6 PUFA, *n*-3 PUFA, and *n*-6/*n*-3 PUFA intake ratio did not affect the prevalence of hypo-HDL-cholesterolemia, even though dietary *n*-3 PUFA has been known to have a beneficial effect on lipid profiles and CVD in previous studies [10,11,12,13,14,15]. The reason that *n*-3 PUFA intake showed no association with hypo-HDL-cholesterolemia in this study may be due to the low *n*-3 PUFA intake of the study participants. Dietary Reference Intakes for Koreans 2015 (KDRI) recommends consuming *n*-3 fatty acids about 1% of total energy intake. However, more than 70% of the participants had consumed *n*-3 PUFA less than 0.8% of total energy intake, which might not be enough to induce beneficial effects. Dietary *n*-6 PUFA also showed no association with hypo-HDL-cholesterolemia in this study. Whether *n*-6 PUFA intake lowers or increases the risk of CVD is still controversial [46,47], but a recent meta-analysis concluded that the intake of *n*-6 PUFA had a neutral effect on the incidence of CVD [48]. In addition, no association between total PUFA intake and hypo-HDL-cholesterolemia may be due to the high proportion of *n*-6 PUFA intake out of total PUFA intake among the study participants. A recent meta-analysis of 20 RCTs have shown that the total PUFA intake had little effect on the serum HDL-cholesterol level, and the effect of the total PUFA intake on CVD remains unclear [49]. Since the *n*-6 PUFA and *n*-3 PUFA are competitively absorbed and metabolized, excessive intake of *n*-6 PUFA can inhibit the bioavailability of *n*-3 PUFA [50]. Therefore, both the absolute intakes of *n*-6 PUFA and *n*-3 PUFA and the intake ratio between these PUFAs are important [51]. Thus, we analyzed the association between *n*-6/*n*-3 PUFA intake ratio and hypo-HDL-cholesterolemia, but no significant association was observed.

In this study, the higher the total flavonoid intakes, the lower the prevalence of hypo-HDL-cholesterolemia in men with high *n*-3 PUFA intakes. However, this association was not observed in men with low *n*-3 PUFA intakes. Our findings of the effect modification of *n*-3 PUFA can be partly explained by the potential synergistic relationship between dietary flavonoids and *n*-3 PUFAs, suggested from animal studies [13,14,15]. For example, flavonoid intake was shown to promote *n*-3 PUFA biosynthesis [13]. *n*-3 PUFA intake also enhanced the bioavailability of flavonoids in animal studies [14,15]. Taken together, we hypothesize that *n*-3 PUFA may enhance the level and function of flavonoids, leading to increased HDL-cholesterol levels. Although the interacting effect of flavonoids and *n*-3 PUFAs on HDL-cholesterol levels appears plausible, a previous RCT found that the interaction between polyphenol intake and *n*-3 PUFA intake from seafood did not affect HDL-cholesterol levels in the subjects with a high risk of CVD [52]. However, this RCT analyzed the intakes of polyphenols and *n*-3 PUFA only from seafood, rather than the total intakes of flavonoids and *n*-3 PUFA. Another recent RCT did not identify the significant synergistic effect of docosahexaenoic acid (DHA) and anthocyanidins on HDL-cholesterol levels [53]. These results may differ from our results because they used bioactive enriched foods and a small number of subjects with metabolic syndrome. Therefore, large-scale cohort studies are needed to confirm the results of the present study.

The inverse relationship between dietary proanthocyanidins with hypo-HDL-cholesterolemia, and the modifying effect of *n*-3 PUFA on the association between total flavonoid intake with hypo-HDL-cholesterolemia were found in men, but not in women. This may have resulted from sex differences in HDL-cholesterol level and oxidative stress level. According to previous studies, women have been known to have higher HDL-cholesterol levels than men [19,20,21]. This sex difference in lipid profile is thought to be the result of a complex network of sex hormones with genetic, inflammatory, and immune factors [54,55]. It is possible that this complex interaction, still not clarified, may have made the sex differences in association with total flavonoid and *n*-3 PUFA intake with hypo-HDL-cholesterolemia. Additionally, women are less susceptible to oxidative stress than men owing to sex hormones, nicotinamide adenine dinucleotide phosphate oxidase (NADPH-oxidase) activity, and other unknown mechanisms [56,57]. Women subjects in the present study also had a healthier lifestyle that might lower oxidative stress compared to men subjects, such as less smoking, less drinking, and higher intake of total flavonoid. Since women have originally lower levels of oxidative stress and higher levels of HDL-cholesterol than men, it is possible that the increase in HDL-cholesterol levels was not observed with increasing intakes of total flavonoid and *n*-3 PUFA among women.

This study had several limitations of note. First, this was a cross-sectional study, and therefore the causal relationship could not be confirmed. Second, dietary intake data were based on a single 24-h recall and, therefore, may not reflect the usual intakes of the subjects. Additionally, the flavonoid intakes might have been underestimated due to incomplete coverage of the flavonoid database (77.2% of the food intake reported in the KNHANES 2013–2016 24-h recall data); however, since the database covers 93.0% of fruit intake and 84.8% of vegetable intake reported in the KNHANES 2013–2016 24-h recall data, the error in the estimated total flavonoid intake from foods would not have been significant. The flavonoid intakes from dietary supplements were not available in the flavonoid database and could not be included in the analyses. Despite these limitations, to the best of our knowledge, this was the only study to report a significant effect modification of the *n*-3 PUFA intake on the association between the total flavonoid intake and the hypo-HDL-cholesterolemia. In addition, our results were strengthened by the use of a nationally representative sample and the systematically constructed flavonoid database.

## 5. Conclusions

In conclusion, total flavonoid intake was not associated with hypo-HDL-cholesterolemia, but the prevalence of hypo-HDL-cholesterolemia was low in men who consumed high amounts of proanthocyanidins. This finding may support the beneficial effects of dietary proanthocyanidins on improving CVD risk factors. We also found that an increased total flavonoid intake correlated with a decreased prevalence of hypo-HDL-cholesterolemia in men with a higher *n*-3 PUFA intake, as well as in men with a lower *n*-6/*n*-3 PUFA intake ratio. These results of this study imply that *n*-3 PUFA intake may modify the association between the total flavonoid intake and the hypo-HDL-cholesterolemia. Additional large prospective studies or clinical trials are needed to confirm the causality of these findings.

## Figures and Tables

**Table 1:** General characteristics of the study subjects according to dietary total flavonoid intake tertile.

	Men				Women			
	Total Flavonoid Intake			*p*-Value ^1^	Total Flavonoid Intake			*p*-Value
	T1 (*n* = 1353)	T2 (*n* = 1354)	T3 (*n* = 1354)		T1 (*n* = 2088)	T2 (*n* = 2089)	T3 (*n* = 2088)	
Age, years, mean ± SE	36.1 ± 0.4	38.7 ± 0.3	41.3 ± 0.4	<0.0001	36.4 ± 0.3	39.7 ± 0.3	42.7 ± 0.3	<0.0001
BMI, kg/m^2^, *n* (%)								
<18.5	39 (2.9)	29 (2.3)	42 (3.3)	0.1157	168 (9.1)	148 (7.9)	121 (6.4)	0.0084
18.5 to <25	781 (57.3)	794 (58.3)	841 (61.7)		1427 (68.3)	1489 (72.0)	1530 (73.2)	
≥25	533 (39.9)	531 (39.3)	471 (35.0)		493 (22.6)	452 (20.1)	437 (20.4)	
Household income, *n* (%)								
Low	128 (9.9)	90 (6.7)	89 (6.8)	<0.0001	180 (8.9)	158 (7.8)	158 (7.7)	<0.0001
Middle-low	352 (26.0)	329 (24.4)	275 (20.4)		575 (27.9)	505 (23.5)	431 (19.5)	
Middle-high	460 (34.0)	446 (32.7)	428 (32.0)		695 (32.8)	669 (31.9)	658 (32.5)	
High	408 (30.1)	484 (36.3)	559 (40.8)		628 (30.4)	753 (36.8)	832 (40.3)	
Education level, *n* (%)								
≤Middle school	149 (9.4)	140 (9.1)	148 (9.2)	<0.0001	274 (11.5)	295 (13.3)	346 (14.8)	0.0838
High school	576 (48.9)	509 (42.5)	447 (37.4)		811 (43.0)	797 (41.2)	800 (40.6)	
≥College	527 (41.7)	602 (48.4)	672 (53.4)		909 (45.5)	882 (45.5)	844 (44.5)	
Alcohol consumption ^2^, *n* (%)								
None or low	271 (20.2)	307 (23.5)	356 (27.2)	<0.0001	922 (43.7)	965 (45.8)	1169 (54.9)	<0.0001
Moderate	298 (24.0)	319 (24.8)	374 (29.1)		509 (24.6)	577 (28.6)	512 (25.4)	
High	412 (32.7)	403 (30.7)	367 (28.2)		415 (22.4)	366 (19.1)	303 (16.7)	
Very high	316 (23.2)	273 (21.0)	208 (15.5)		191 (9.3)	126 (6.5)	56 (3.0)	
Smoking status ^3^, *n* (%)								
Never	362 (30.4)	383 (30.5)	428 (35.7)	<0.0001	1771 (86.1)	1844 (90.0)	1919 (93.4)	<0.0001
Former	264 (18.6)	358 (25.8)	444 (30.8)		113 (5.9)	80 (4.0)	69 (3.7)	
Current	671 (51.0)	561 (43.7)	433 (33.5)		153 (8.0)	110 (6.1)	52 (2.9)	
Physical activity ^4^, *n* (%)								
No	573 (43.4)	564 (42.3)	554 (42.0)	0.7571	1107 (53.2)	1038 (50.1)	1023 (49.9)	0.1245
Yes	676 (56.6)	687 (57.7)	712 (58.0)		886 (46.8)	937 (49.9)	964 (50.1)	
Fasting blood cholesterol and triglyceride levels, mean ± SE								
Total cholesterol, mg/dL	189.6 ± 1.0	188.6 ± 1.0	192.4 ± 1.1	0.0293	184.9 ± 0.8	186.6 ± 0.8	190.6 ± 0.9	<0.0001
Triglycerides, mg/dL	157.8 ± 3.9	160.6 ± 4.1	150.3 ± 3.6	0.1211	99.5 ± 1.7	98.2 ± 1.6	99.0 ± 1.6	0.8469
HDL-cholesterol, mg/dL	47.8 ± 0.3	48.1 ± 0.3	48.2 ± 0.3	0.6661	56.4 ± 0.3	56.3 ± 0.3	56.0 ± 0.3	0.6651
LDL-cholesterol ^5^, mg/dL	112.3 ± 0.9	111.4 ± 0.9	116.1 ± 0.9	0.0006	109.0 ± 0.7	110.8 ± 0.7	114.9 ± 0.7	<0.0001
Nutrient intakes ^6^, mean ± SE								
Energy, Kcal/d	2503.6 ± 25.7	2560.7 ± 25.9	2378.8 ± 24.7	<0.0001	1828.3 ± 17.7	1858.9 ± 16.3	1736.0 ± 15.5	<0.0001
Carbohydrate, g/d	319.7 ± 2.3	328.3 ± 2.2	353.6 ± 2.1	<0.0001	250.3 ± 1.3	259.4 ± 1.3	276.0 ± 1.3	<0.0001
Protein, g/d	80.6 ± 0.7	84.3 ± 0.7	82.5 ± 0.7	0.0021	59.3 ± 0.5	60.3 ± 0.4	58.3 ± 0.4	0.0049
Fat, g/d	55.4 ± 0.7	55.0 ± 0.7	52.3 ± 0.7	0.0024	41.7 ± 0.4	40.3 ± 0.4	37.4 ± 0.5	<0.0001
Total flavonoids, mg/d	41.1 ± 0.6	117.3 ± 0.9	453.6 ± 14.2	<0.0001	38.7 ± 0.5	129.1 ± 0.9	463.1 ± 10.2	<0.0001
Flavonols, mg/d	15.3 ± 0.3	28.2 ± 0.6	42.9 ± 2.1	<0.0001	12.3 ± 0.2	21.0 ± 0.5	33.9 ± 1.4	<0.0001
Flavones, mg/d	0.9 ± 0.0	1.2 ± 0.0	2.3 ± 0.6	<0.0001	0.8 ± 0.0	1.0 ± 0.0	1.6 ± 0.2	<0.0001
Flavanones, mg/d	0.9 ± 0.1	6.4 ± 0.6	17.0 ± 1.5	<0.0001	1.7 ± 0.2	11.5 ± 0.8	20.6 ± 1.9	<0.0001
Flavan-3-ols, mg/d	1.6 ± 0.1	5.4 ± 0.3	62.9 ± 6.9	<0.0001	1.9 ± 0.1	8.7 ± 0.4	68.9 ± 6.5	<0.0001
Isoflavones, mg/d	9.1 ± 0.3	20.0 ± 0.7	29.1 ± 1.4	<0.0001	7.4 ± 0.2	15.1 ± 0.5	19.4 ± 1.0	<0.0001
Anthocyanidins, mg/d	9.9 ± 0.5	36.3 ± 1.6	113.9 ± 6.5	<0.0001	9.7 ± 0.3	35.5 ± 1.1	115.1 ± 5.8	<0.0001
Proanthocyanidins, mg/d	4.6 ± 0.3	25.5 ± 1.6	282.1 ± 17.7	<0.0001	6.2 ± 0.3	40.8 ± 1.3	286.1 ± 10.5	<0.0001
Total PUFA, g/d	11.8 ± 0.2	13.7 ± 0.2	13.4 ± 0.2	<0.0001	9.3 ± 0.1	10.1 ± 0.1	9.7 ± 0.2	<0.0001
*n*-6 PUFA, g/d	10.4 ± 0.2	11.9 ± 0.2	11.5 ± 0.2	<0.0001	8.1 ± 0.1	8.7 ± 0.1	8.2 ± 0.1	0.0003
*n*-3 PUFA, g/d	1.5 ± 0.0	1.9 ± 0.0	1.9 ± 0.0	<0.0001	1.2 ± 0.0	1.4 ± 0.0	1.5 ± 0.0	<0.0001
*n*-6/*n*-3 PUFA	9.6 ± 0.2	8.0 ± 0.1.0	7.7 ± 0.1	<0.0001	8.9 ± 0.1	8.1 ± 0.1	7.4 ± 0.1	<0.0001

For all values, the PROC SURVEY procedure was used to account for the complex sampling design effect of the national surveys; Values are presented as means ± standard errors for continuous variables and as frequencies (percentages) for categorical variables; ^1^ All continuous variables were tested using the analysis of variance (ANOVA) and all categorical variables were tested using the chi-square test; ^2^ Alcohol consumption was defined as none or low (less than once per month for the last year), moderate (less than twice per week and an average consumption per event of is <7 cups for men and <5 cups for women), high (more than once per month but not moderate nor very high drinker), and very high (more than twice per week and an average consumption per event of ≥7 cups for men and ≥5 cups for women); ^3^ Smoking status was defined as never (never smoked cigarettes or smoked <100 cigarettes during lifetime), former (smoked ≥100 cigarettes during lifetime but currently non-smoking), and current (smoked ≥100 cigarettes during lifetime and currently smoking); ^4^ Physical activity was recorded as “yes” if the participant stated that they performed activity at a vigorous intensity for >75 min per week or at a moderate intensity for >150 min per week, or an equivalent combination of moderate and vigorous activity; ^5^ LDL-cholesterol was calculated according to Friedewald’s formula (when the triglycerides level <400 mg/dL): (LDL-cholesterol) = (total cholesterol) - (HDL-cholesterol) - (triglyceride/5); ^6^ Dietary intakes of nutrients were adjusted for energy intake using the residual method; Missing values: men - 13 for household income, 291 for education level, 157 for alcohol consumption, 157 for smoking status, 295 for physical activity, and 156 for LDL-cholesterol, women - 23 for household income, 307 for education level, 154 for alcohol consumption, 154 for smoking status, 310 for physical activity, and 42 for LDL-cholesterol; Abbreviations: HDL: high-density lipoprotein; LDL: low-density lipoprotein; BMI: body mass index; PUFA: polyunsaturated fatty acid; T1, T2, and T3: tertile 1, 2 and 3; SE: standard error.

**Table 2:** Odds ratios and 95% confidence intervals for hypo-HDL-cholesterolemia in Korean adults according to the flavonoid and polyunsaturated fatty acid intake tertile ^1,2,3^.

	Men				Women			
	T1	T2	T3	*p* for Trend	T1	T2	T3	*p* for Trend
Total flavonoid	1.00 (ref)	1.07 (0.86–1.33)	0.85 (0.68–1.07)	0.0788	1.00 (ref)	1.11 (0.94–1.30)	1.04 (0.89–1.22)	0.8835
Flavonols	1.00 (ref)	1.15 (0.93–1.43)	1.02 (0.82–1.26)	0.9565	1.00 (ref)	1.06 (0.91–1.25)	1.05 (0.90–1.22)	0.6386
Flavones	1.00 (ref)	1.07 (0.86–1.31)	0.97 (0.78–1.20)	0.6407	1.00 (ref)	1.05 (0.90–1.24)	1.14 (0.97–1.34)	0.1036
Flavanones	1.00 (ref)	0.93 (0.75–1.15)	0.88 (0.71–1.09)	0.3346	1.00 (ref)	1.03 (0.87–1.22)	0.99 (0.84–1.16)	0.7083
Flavan-3-ols	1.00 (ref)	0.99 (0.79–1.23)	0.91 (0.72–1.16)	0.4161	1.00 (ref)	0.81 (0.68–0.96)	1.03 (0.87–1.22)	0.1413
Isoflavones	1.00 (ref)	0.88 (0.71–1.10)	0.98 (0.80–1.21)	0.8766	1.00 (ref)	1.05 (0.90–1.23)	0.95 (0.81–1.12)	0.3849
Anthocyanidins	1.00 (ref)	0.99 (0.79–1.23)	1.07 (0.85–1.34)	0.5027	1.00 (ref)	0.89 (0.76–1.05)	1.05 (0.90–1.23)	0.2299
Proanthocyanidins	1.00 (ref)	0.81 (0.64–1.02)	0.74 (0.59–0.92)	0.0330	1.00 (ref)	0.99 (0.84–1.17)	0.95 (0.81–1.12)	0.5240
Total PUFA	1.00 (ref)	0.86 (0.69–1.08)	0.93 (0.75–1.16)	0.6621	1.00 (ref)	0.99 (0.84–1.16)	0.90 (0.76–1.06)	0.1715
*n*-6 PUFA	1.00 (ref)	0.81 (0.65–1.00)	0.92 (0.74–1.14)	0.5850	1.00 (ref)	0.97 (0.82–1.14)	0.88 (0.74–1.04)	0.1153
*n*-3 PUFA	1.00 (ref)	0.90 (0.73–1.12)	1.08 (0.86–1.34)	0.3713	1.00 (ref)	1.02 (0.87–1.21)	1.00 (0.86–1.17)	0.9890
*n*-6/*n*-3 PUFA intake ratio	1.00 (ref)	0.98 (0.79–1.23)	0.87 (0.69–1.09)	0.2199	1.00 (ref)	0.93 (0.79–1.09)	0.88 (0.75–1.02)	0.0843

^1^ Dietary intakes of flavonoids and polyunsaturated fatty acids were adjusted for energy intake using the residual method; ^2^ Odds ratios and 95% confidence intervals were adjusted for age, BMI, serum triglyceride level, alcohol consumption, smoking status, household income, education level, and energy intake; ^3^ Hypo-HDL-cholesterolemia was defined as serum HDL-cholesterol <40 mg/dL for men and <50 mg/dL for women; Abbreviations: HDL: high-density lipoprotein; OR: odds ratio; CI: confidence interval; ref: reference; PUFA: polyunsaturated fatty acid; T1, T2, and T3: tertile 1, 2 and 3.

**Table 3:** Odds ratios and 95% confidence intervals for hypo-HDL-cholesterolemia in Korean adults according to the total flavonoid intake tertile after stratification by the dietary polyunsaturated fatty acid intake ^1,2,3.^

	Men					Women				
	Total Flavonoid Intake			*p* for Trend	*p* for Interaction ^4^	Total Flavonoid Intake			*p* for Trend	*p* for Interaction
	T1	T2	T3			T1	T2	T3		
Total PUFA intake										
Low (<median)	1.00 (ref)	1.09 (0.80–1.47)	0.94 (0.68–1.28)	0.5505	0.5494	1.00 (ref)	1.07 (0.86–1.34)	0.88 (0.70–1.10)	0.1482	0.1785
High (≥median)	1.00 (ref)	1.07 (0.79–1.46)	0.79 (0.56–1.11)	0.0822		1.00 (ref)	1.16 (0.91–1.47)	1.25 (0.98–1.59)	0.0987	
*n*-6 PUFA intake										
Low (<median)	1.00 (ref)	1.07 (0.79–1.44)	0.88 (0.65–1.20)	0.3144	0.8782	1.00 (ref)	1.12 (0.89–1.41)	0.92 (0.74–1.15)	0.2835	0.3247
High (≥median)	1.00 (ref)	1.09 (0.80–1.50)	0.86 (0.60–1.21)	0.2270		1.00 (ref)	1.10 (0.87–1.40)	1.18 (0.92–1.50)	0.2103	
*n*-3 PUFA intake										
Low (<median)	1.00 (ref)	1.16 (0.86–1.59)	1.19 (0.87–1.63)	0.3258	0.0038	1.00 (ref)	1.19 (0.96–1.48)	0.98 (0.78–1.24)	0.6220	0.3757
High (≥median)	1.00 (ref)	0.93 (0.68–1.26)	0.59 (0.42–0.82)	0.0004		1.00 (ref)	1.05 (0.83–1.33)	1.10 (0.87–1.39)	0.4645	
*n*-6/*n*-3 PUFA intake ratio										
Low (<median)	1.00 (ref)	0.97 (0.71–1.33)	0.67 (0.48–0.93)	0.0053	0.1772	1.00 (ref)	1.06 (0.85–1.34)	1.00 (0.80–1.25)	0.8256	0.9475
High (≥median)	1.00 (ref)	1.12 (0.83–1.51)	1.06 (0.77–1.44)	0.8344		1.00 (ref)	1.14 (0.92–1.42)	1.06 (0.84–1.34)	0.7935	

^1^ Dietary intakes of total flavonoid and polyunsaturated fatty acids were adjusted for energy intake using the residual method; ^2^ Odds ratios and 95% confidence intervals were adjusted for age, BMI, serum triglyceride level, alcohol consumption, smoking status, household income, education level, and energy intake; ^3^ Hypo-HDL-cholesterolemia was defined as serum HDL-cholesterol <40 mg/dL for men and <50 mg/dL for women; ^4^ *p* for interaction values were obtained by Wald tests of the cross-product of total flavonoids intake categories (tertile 1, 2 and 3) and polyunsaturated fatty acids intake categories (<median, ≥median) (all ordinal variables; 1 degree of freedom); Median intakes of PUFA: men—11.4 g/d for total PUFA, 9.8 g/d for *n*-6 PUFA, 1.4 g/d for *n*-3 PUFA, and 7.7 for *n*-6/*n*-3 PUFA intake ratio; women—8.5 g/d for total PUFA, 7.3 g/d for n-6 PUFA, 1.1 g/d for *n*-3 PUFA, and 7.4 for *n*-6/*n*-3 PUFA intake ratio; Abbreviations: HDL: high-density lipoprotein; OR: odds ratio; CI: confidence interval; ref: reference; PUFA: polyunsaturated fatty acid; T1, T2, and T3: tertile 1,2, and 3.

## Supplementary Materials

The following are available online at https://www.mdpi.com/2072-6643/12/1/195/s1, Table S1: Odds ratios and 95% confidence intervals for hypo-HDL-cholesterolemia in Korean adults according to the proanthocyanidins intake tertile after stratification by the dietary polyunsaturated fatty acid intake.
