# Supplementary material for: Association of Total Flavonoid Intake with Hypo-HDL-Cholesterolemia among Korean Adults: Effect Modification by Polyunsaturated Fatty Acid Intake
Source: Nutrients. 2020 Jan 10;12(1):195. doi: 10.3390/nu12010195 (PMC7019369; doi:10.3390/nu12010195)
Supplement: Supplementary file 1 [file nutrients-12-00195-s001.pdf]

Supplementary Table S1. Odds ratios and 95% confidence intervals for hypo-HDL-cholesterolemia in Korean adults according to the proanthocyanidins intake tertile after stratification by the dietary polyunsaturated fatty acid intake [1,2,3].

	Men					Women				
	Proanthocyanidins Intake			*p* for Trend	*p* for Interaction [4]	Proanthocyanidins Intake			*p* for Trend	*p* for Interaction
	T1	T2	T3			T1	T2	T3		
Total PUFA intake										
Low (<median)	1.00 (ref)	0.76 (0.55-1.03)	0.73 (0.54-1.00)	0.1626	0.6622	1.00 (ref)	1.02 (0.82-1.28)	0.95 (0.76-1.18)	0.5255	0.7890
High (≥median)	1.00 (ref)	0.87 (0.63-1.20)	0.74 (0.54-1.02)	0.0947		1.00 (ref)	0.97 (0.76-1.23)	0.95 (0.76-1.21)	0.7437	
n-6 PUFA intake										
Low (<median)	1.00 (ref)	0.77 (0.57-1.05)	0.72 (0.53-0.97)	0.0935	0.8932	1.00 (ref)	1.01 (0.80-1.27)	0.99 (0.79-1.23)	0.8685	0.8376
High (≥median)	1.00 (ref)	0.85 (0.61-1.17)	0.77 (0.56-1.07)	0.2028		1.00 (ref)	0.99 (0.78-1.25)	0.91 (0.72-1.16)	0.4302	
n-3 PUFA intake										
Low (<median)	1.00 (ref)	0.78 (0.58-1.06)	0.82 (0.60-1.13)	0.5015	0.2360	1.00 (ref)	1.05 (0.84-1.31)	0.92 (0.73-1.16)	0.3363	0.5594
High (≥median)	1.00 (ref)	0.84 (0.62-1.15)	0.66 (0.49-0.89)	0.0120		1.00 (ref)	0.94 (0.74-1.20)	0.98 (0.78-1.24)	0.9608	
n-6/*n*-3 PUFA intake ratio										
Low (<median)	1.00 (ref)	0.79 (0.56-1.10)	0.68 (0.50-0.93)	0.0451	0.9018	1.00 (ref)	1.10 (0.87-1.38)	1.04 (0.84-1.30)	0.9991	0.2878
High (≥median)	1.00 (ref)	0.84 (0.62-1.14)	0.79 (0.57-1.09)	0.2564		1.00 (ref)	0.89 (0.71-1.11)	0.86 (0.68-1.10)	0.3376	

[1] Dietary intakes of proanthocyanidins and polyunsaturated fatty acids were adjusted for energy intake using the residual method; [2] Odds ratios and 95% confidence intervals were adjusted for age, BMI, serum triglyceride level, alcohol consumption, smoking status, household income, education level, and energy intake; [3] Hypo-HDL-cholesterolemia was defined as serum HDL-cholesterol < 40 mg/dL for men and < 50 mg/dL for women; [4] *p* for interaction values were obtained by Wald tests of the cross-product of proanthocyanidins intake categories (tertile 1, 2 and 3) and polyunsaturated fatty acids intake categories (<median, ≥median) (all ordinal variables; 1 degree of freedom); Abbreviations: HDL: high density lipoprotein; OR: odds ratio; CI: confidence interval; ref: reference; PUFA: polyunsaturated fatty acid; T1, T2, and T3: tertile 1, 2 and 3.
